# *In Silico* Repositioning-Chemogenomics Strategy Identifies New Drugs with Potential Activity against Multiple Life Stages of *Schistosoma mansoni*

**DOI:** 10.1371/journal.pntd.0003435

**Published:** 2015-01-08

**Authors:** Bruno J. Neves, Rodolpho C. Braga, José C. B. Bezerra, Pedro V. L. Cravo, Carolina H. Andrade

**Affiliations:** 1 LabMol – Laboratory for Drug Design and Modeling, Faculdade de Farmácia, Universidade Federal de Goiás, Goiânia, Brazil; 2 Instituto de Patologia Tropical e Saúde Pública, Universidade Federal de Goiás, Goiânia, Brazil; 3 Instituto de Química, Universidade Federal de Goiás, Goiaânia, Brazil; 4 Centro de Malária e Doenças Tropicais, Instituto de Higiene e Medicina Tropical, Universidade Nova de Lisboa, Lisboa, Portugal; Khon Kaen University, Thailand

## Abstract

Morbidity and mortality caused by schistosomiasis are serious public health problems in developing countries. Because praziquantel is the only drug in therapeutic use, the risk of drug resistance is a concern. In the search for new schistosomicidal drugs, we performed a target-based chemogenomics screen of a dataset of 2,114 proteins to identify drugs that are approved for clinical use in humans that may be active against multiple life stages of *Schistosoma mansoni*. Each of these proteins was treated as a potential drug target, and its amino acid sequence was used to interrogate three databases: Therapeutic Target Database (TTD), DrugBank and STITCH. Predicted drug-target interactions were refined using a combination of approaches, including pairwise alignment, conservation state of functional regions and chemical space analysis. To validate our strategy, several drugs previously shown to be active against *Schistosoma* species were correctly predicted, such as clonazepam, auranofin, nifedipine, and artesunate. We were also able to identify 115 drugs that have not yet been experimentally tested against schistosomes and that require further assessment. Some examples are aprindine, gentamicin, clotrimazole, tetrabenazine, griseofulvin, and cinnarizine. In conclusion, we have developed a systematic and focused computer-aided approach to propose approved drugs that may warrant testing and/or serve as lead compounds for the design of new drugs against schistosomes.

## Author Summary

Schistosomiasis is a neglected tropical disease caused by schistosome parasites that affects millions of people worldwide. The current reliance on a single drug (Praziquantel) for treatment and control of the disease calls for the urgent discovery of novel schistosomicidal agents. One approach that can expedite drug discovery is to find new uses for existing approved drugs, a practice known as drug repositioning. Currently, modern drug repositioning strategies entail the search for compounds that act on a specific target, often a protein known or suspected to be required for survival of the parasite. Drug repositioning approaches for schistosomiasis are now greatly facilitated by the availability of comprehensive schistosome genome data in user-friendly databases. Here, we report a drug repositioning computational strategy that involves identification of novel schistosomicidal drug candidates using similarity between schistosome proteins and known drug targets. Researchers can now use the list of predicted drugs as a basis for deciding which potential schistosomicidal candidates can be tested experimentally.

## Introduction

Schistosomiasis is one of the main neglected tropical diseases affecting humans. It is caused by flatworms of the genus *Schistosoma* (*S. mansoni*, *S. japonicum*, *S. haematobium*, *S. intercalatum* and *S. mekongi*) [1]–[3]. This parasitic disease ranks second only after malaria in terms of its public health importance [4] because of its chronic and debilitating characteristics that result in a substantial burden on human health [5], [6]. Recent estimates suggest that more than 249 million people were infected in 78 endemic countries located in sub-Saharan Africa, the Middle East, the Caribbean, and South America, resulting in 200,000 deaths annually [7].

Schistosomes have complex life cycles that involve vertebrate (often a mammal) and invertebrate (aquatic snail) hosts, in which sexual and asexual reproductive phases occur, respectively. Mammalian definitive hosts are infected via skin penetration by cercariae, which lose their bifurcated tail and become schistosomula [8], [9]. After 5–7 days, schistosomula migrate from the lungs to the hepatic portal system via the blood stream and transform into adult worms. Male and female worms pair in the hepatic portal system and migrate to the mesenteric veins (except *S. haematobium,* which migrates to the urogenital system) to lay nearly 300 eggs per day. These eggs either pass into the gut lumen to be voided in the faeces and continue the life cycle or pass through the mesenteric veins and lodge in the liver, where they can cause granulomatous changes and fibrosis, both of which are key contributors to schistosomiasis [8], [10].

In the absence of a vaccine, praziquantel (PZQ) has been the drug of choice recommended by the World Health Organization for the treatment and control of all the major *Schistosoma* species in mass drug administration programs for almost three decades [11]. More recently, the use of artemisinin derivatives alone or in combination with PZQ for the treatment and prevention of schistosomiasis has shown encouraging results [12], but it is unlikely to represent an ideal stand-alone drug-based control strategy. Moreover, the suboptimal efficacy of PZQ against immature worms that are present in newly acquired infections [13] and the prospect of drug resistance indicate a need to identify new schistosomicidal drugs active against multiple stages of parasite life cycle [5], [14]–[16].

One approach that can expedite drug discovery process is to find new uses for existing approved compounds, a practice commonly known as drug repositioning or repurposing [17]. Drug repositioning has proved to be an efficient way of identifying new therapies against neglected tropical diseases. A recent example of a repositioned drug is miltefosine, a drug that was originally developed to treat breast cancer and is now used against visceral leishmaniasis [18], [19]. In addition to saving money and time, an advantage of drug repositioning is that the existing drugs have already been scrutinized in terms of pharmacokinetic and toxicity parameters [20]–[22].

Over the last few decades, advances in computer technologies have resulted in useful tools to assist early drug discovery and development. In this context, the use of *in silico* tools can reduce the cost and the time required to select the most promising candidates for *in vitro* and *in vivo* assays [20]. Our laboratory has been developing and applying many computer-assisted drug discovery (CADD) strategies in the hope of discovering new drug candidates for neglected tropical diseases [23]–[43].

Several *in silico* chemogenomic studies have demonstrated that genome-wide gene expression data might also represent a useful resource for identifying drugs and drug target genes that can potentially be used for drug repositioning [44]–[47]. The ultimate goal of chemogenomics is to establish the molecular relationship(s) between ligands and drug targets. Therefore, various publicly available databases, such as Therapeutic Target Database (TTD) [48], DrugBank [49], and STITCH [50], which integrate information about gene/protein–drug–disease interactions, are useful resources to develop these strategies. Based on the concept that “similar targets have similar ligands”, homology-based searching using these databases helps to identify compounds that may act on a target for which there are no known active compounds but that are related by homology to one or more targets for which active compounds are known [51], [52]. In such context, *S. mansoni* targets with structural homology similar to known targets of approved drugs are more likely to be susceptible to inhibitors listed in the drug target and drug databases.

Recently, Protasio et al. [53] used transcriptomic sequencing from four time points in the *S. mansoni* life cycle to refine gene predictions and establish expression profiles in the parasite. Consequently, a high-resolution map of the temporal changes in the transcription of genes was produced for all intra-mammalian life cycle stages of *S. mansoni*. These data have been compiled into a searchable format within the SchistoDB (www.schistodb.net) and GeneDB (www.genedb.org) databases [53]. Transcription profiling and genome sequencing data provide important fundamental information to support further advances in schistosome research. In the present study, we used an *in silico* target-based chemogenomics strategy, integrating *S. mansoni* genomics data with drug-target database resources to predict new drugs with potential activity against multiple life stages of *S. mansoni*.

## Materials and Methods

### Compilation of the list of *S. mansoni* genes

**Figure 1:**
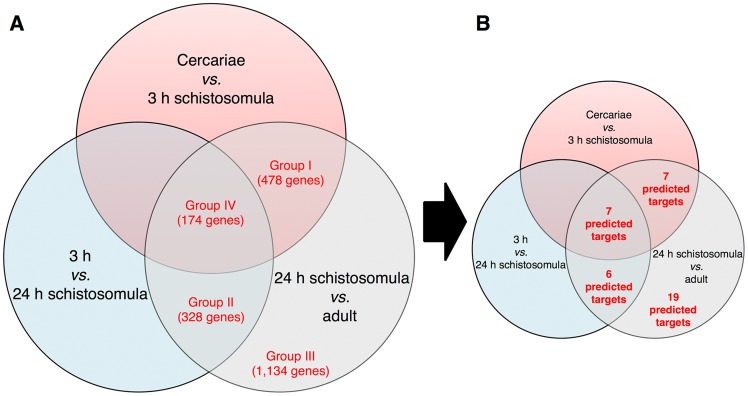
Distribution of genes from *S. mansoni* and predicted targets. (**A**) Venn diagram represents the clustering of genes in four different groups according to temporal life stages of the parasite. (**B**) Rate of druggable *S. mansoni* targets identified for each group using the target-based chemogenomics approach.

**Figure 2:**
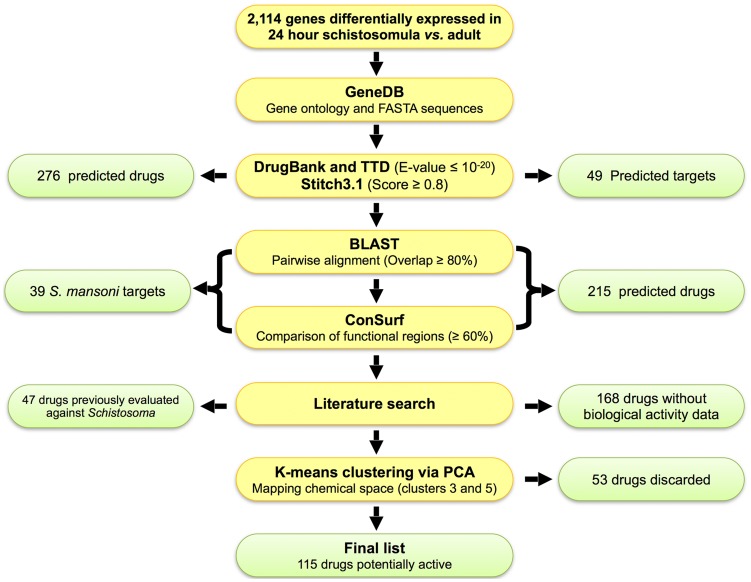
Flowchart summarizing the *in silico* repositioning chemogenomics strategy and corresponding results. The green boxes represent the summarized results obtained at each stage of the study.

The target-based chemogenomics screening was performed on a dataset containing 2,076 genes that are differentially expressed among the 24 hour schistosomula *vs.* adult life stages obtained from Protasio et al. [53]. We also obtained 38 *S. mansoni* genes from the TDR Targets database [54] using the target search tool. We searched for targets with “any form of validation”, which included “genetic”, “pharmacological”, and “observed phenotypes” (S1 Table). We focused on searching for genes that are expressed in “24 h schistosomula *vs.* adult” because they are intra-mammalian stages. However, some of these genes are also expressed in other temporal life cycle stages, such as “cercariae *vs*. 3 hour schistosomula” and “3 h *vs.* 24 h schistosomula”. These genes are considered promising targets for prophylactic drugs because they are involved in the penetration through the mammalian host's skin, host adaptation, and differentiation and growth of the parasite. Therefore, genes were grouped according to the following division: group I was composed of genes differentially transcribed between “24 h schistosomula *vs.* adult” and also between “cercariae and 3 h schistosomula”; group II was composed of genes differentially transcribed between “24 h schistosomula *vs.* adults” as well as between “3 h and 24 h schistosomula”; group III was composed of genes transcribed between “24 h schistosomula *vs.* adult”; and group IV was composed of genes transcribed concurrently in all the life cycle stages (Fig. 1A). Information for individual genes or gene products (primary amino acid sequence in FASTA format, target name, and biological process/es) was then retrieved from the GeneDB *S. mansoni* genome database [55]. We then verified the annotation of each single putative protein and corrected it, if necessary, according to the recent updated annotations in the GeneDB database. For convenience, the *in silico* target-based chemogenomic pipeline is presented in Fig. 2.

### Identification of putative drug targets using publicly available drug databases

Each predicted protein from *S. mansoni* was used to interrogate three different publicly available databases that provide detailed information on drugs and their targets: TTD [48], DrugBank [49], and STITCH [50]. The search strategy for DrugBank and TTD was based on the principle of homology, whereby each query (*S. mansoni* target) was compared for matches to known drug targets contained in each of these databases. In cases where homologous drug targets were identified, all proteins with an output expectation value (E-value) of ≤10^−20^ for DrugBank and TTD were listed as “acceptable targets”. This E-value represents the number of hits with an alignment score “Z” or equal or better than “Z” that would be expected by chance when searching a database. The E-value is the expected number of times a homology match will occur at random in a given set of trials. However, the STITCH database integrates data from the literature and various databases of biological pathways, drug–target relationships, and binding affinities. The resultant network can be explored interactively or used as the basis for a confidence score ranging from 0–1. The confidence score is a set of high-confidence interactions between drugs and targets (i.e., proteins with shared Gene Ontology annotations) that is used as a reference for screening results. Therefore, in the case of the STITCH database, when significant matches were found, only targets with a score ≥0.8 were considered [50]. We filtered all predicted targets through inclusion in the list of only those proteins that were indicated to interact with approved drugs, excluding the nutraceutical class, as these compounds are unlikely to exhibit schistosomicidal activity [56].

### Pairwise alignment and comparison of functional regions

Predicted *S. mansoni* targets were aligned with their homologue drug targets using pairwise BLAST [57]. We considered the *S. mansoni* targets for further evaluation only in cases where there was ≥80% overlap with the corresponding drug target. This filtering step enhances the probability of both proteins sharing the same active site. Subsequently, we performed the sequence alignment and compared the functional regions among the approved drug targets and *S. mansoni* targets using the ConSurf server [58]. This procedure was used to estimate the conservation of active sites between the proteins and the preservation of affinity for the predicted schistosomicidal drugs. The ConSurf server [58] is a bioinformatic tool for estimating the evolutionary conservation of amino acid positions in a protein based on the phylogenetic relationships between homologous sequences. Therefore, the degree of conservation of the amino acids within the active site of each approved drug target was estimated using 150 homologues from other organisms with similar sequences in the UniProt database [59]. The sequences were clustered and those presenting high sequence similarity (>95%) were excluded using the algorithm CD-HIT [60] to filter out redundant sequences. In the same way, the sequences that shared less than the given identity cutoff of <35% were also ignored [58]. A multiple sequence alignment (MSA) of the homologous sequences was constructed using the MAFFT-L-INS-I method [61], and the phylogenetic tree was built using the neighbor-joining algorithm [62]. Position-specific conservation scores were computed using the empirical Bayesian method [63]. Next, the functional regions of each approved drug target were visually compared with the corresponding *S. mansoni* target. The results were classified as functional residues with high (≥80%) or moderate conservation (60–79%). In cases where the conservation between functional residues was less than 59%, the putative targets were excluded from further analyses.

### List of drugs yet to be tested against *Schistosoma* species

We carried out a literature search using the PubMed, PubChem Bioassay database, and SciFinder engines to identify approved drugs that have not been evaluated against *Schistosoma* species by querying all predicted drugs previously identified. The PubChem BioAssay database reports the available biological screening results for the chemical compounds described in the PubChem database, providing searchable descriptions of each bioassay, including descriptions of the conditions and readouts specific to that screening procedure. SciFinder is a chemistry research application that provides access to the world's most comprehensive and authoritative sources of references, chemical substances, and reactions in chemistry and is updated daily by Chemical Abstracts Service. Our definition of “evaluation” embraces biochemical or *in vitro* and *in vivo* assessments of one or more life stages of *Schistosoma* species. Therefore, if a given drug is noted as “not tested”, it means that no publication record was found after either of the following search details: 1. (“drug name” [MeSH Terms] OR “drug name” [All Fields]) AND (“*Schistosoma”* [MeSH Terms] OR “*Schistosoma*” [All Fields]) and 2. (“drug name” [MeSH Terms] OR “drug name” [All Fields]) AND (“schistosomiasis” [MeSH Terms] OR “schistosomiasis” [All Fields]). It also might mean that the studies/assays retrieved were insufficiently informative to infer the potential usefulness of the drug as a schistosomicidal drug or lead compound.

### Chemical space analysis

We evaluated the “chemical space” of known active and inactive compounds against *Schistosoma*. The aim in using this strategy is to find whether predicted compounds share essential structural and physicochemical properties with schistosomicidal compounds. Initially, a dataset of active compounds with enzymatic, *in vitro*, and/or *in vivo* activity data for *Schistosoma* species was collected from the literature (S2 Table) using the PubMed and PubChem Bioassay databases [64]. Because of the differences in the experiments used for the biological activity evaluation, compounds of our database were considered to be active according to the specifications of each study or bioassay. In addition, a dataset of inactive compounds was compiled from a large dataset of non-inhibitors of the enzyme thioredoxin glutathione reductase of *S. mansoni* previously reported (PubChem Bioassay AID: 485364). These compounds were assumed to be inactive, because in the literature they were not reported to produce any schistosomicidal activity. All aforementioned chemical datasets were carefully curated and standardized according to the protocol proposed by Fourches et al. [65]. Structural normalization of specific chemotypes, such as aromatic and nitro groups, was performed using ChemAxon Standardizer (v.6.1.3, ChemAxon, Budapest, Hungary, http://www.chemaxon.com). Duplicates (i.e., identical compounds reported several times in the dataset) were identified using KSAR workflow (http://labmol.farmacia.ufg.br/ksar). The dataset is unbalanced, meaning that the size of the active and inactive classes does not match. Therefore, we used the algorithm *k*-Nearest Neighbor (*k*NN) developed in software R and the qsaR 1.5 package (http://labmol.farmacia.ufg.br/chemoinformatics) to equalize the number of compounds in different classes; this is referred to as “dataset balancing”. The basic principle here is to evaluate the whole active dataset represented by the MACCS fingerprint matrix evaluating the Euclidean distance to the MACCS fingerprint of each inactive compound. The compounds were reordered by nearest *k*-distance of the active compounds. Thereafter, a set representing 39 descriptors accounting for physicochemical properties were calculated using RDKit 2.4.0 [66]. The descriptor matrix was normalized, and low variance descriptors (variance upper bound set to 0.0) were removed before generating the model. The chemical space analysis of predicted drugs was generated using *k*-means clustering space using Principal Component Analysis (PCA) and employing the software R v.3.0.3 [67]. All steps of dataset balancing, processing, and chemical space analysis were implemented in R and KNIME, a graphical user interface that allows the assembly of nodes for modeling, data analysis, and visualization (S1 Fig.).

## Results

### Compilation of the genes list

The dataset of *S. mansoni* genes was compiled from the Protasio et al. study and the TDR Targets database, totalling 2,114 genes (S1 Table). We focused on searching drugs with potential activity in schistosomula and adult life cycle stages, which are all intra-mammalian stages. For this reason, genes that do not have a differential transcription between 24 h schistosomula and adults were not considered. Some of the genes differentially transcribed between these stages were also transcribed in other life cycle stages, including the cercariae; therefore, some of these genes are considered promising targets for drugs, as they are expected to be involved in penetration through mammalian host skin, adaptation, differentiation, and growth. The 2,114 genes were clustered in four main groups (I–IV) according to transcription in each life cycle stage (Fig. 1A). Totals of 478, 328, 1,134, and 174 transcribed genes were identified.

### Identification of putative drug targets using publicly available drug databases

The information about individual genes (primary amino acid sequence in FASTA format, target name, and biological process) was retrieved from the GeneDB *S. mansoni* genome database. Based on the FASTA sequence information, we predicted schistosomicidal drugs using the sequence similarity screening in three databases (DrugBank, STITCH 3.1, and TTD). In this step, numerical statistical probability parameters (E-value ≤10^−20^ or a score ≥0.8) were adopted to provide high confidence for the data. We decided to use all three databases because each of them may contain different drug-target datasets and, consequently, the probability of targets and their drugs being missed is reduced. This analysis predicted 49 targets associated with 276 approved drugs (S3 Table).

### Pairwise alignment and comparison of functional regions

**Figure 3:**
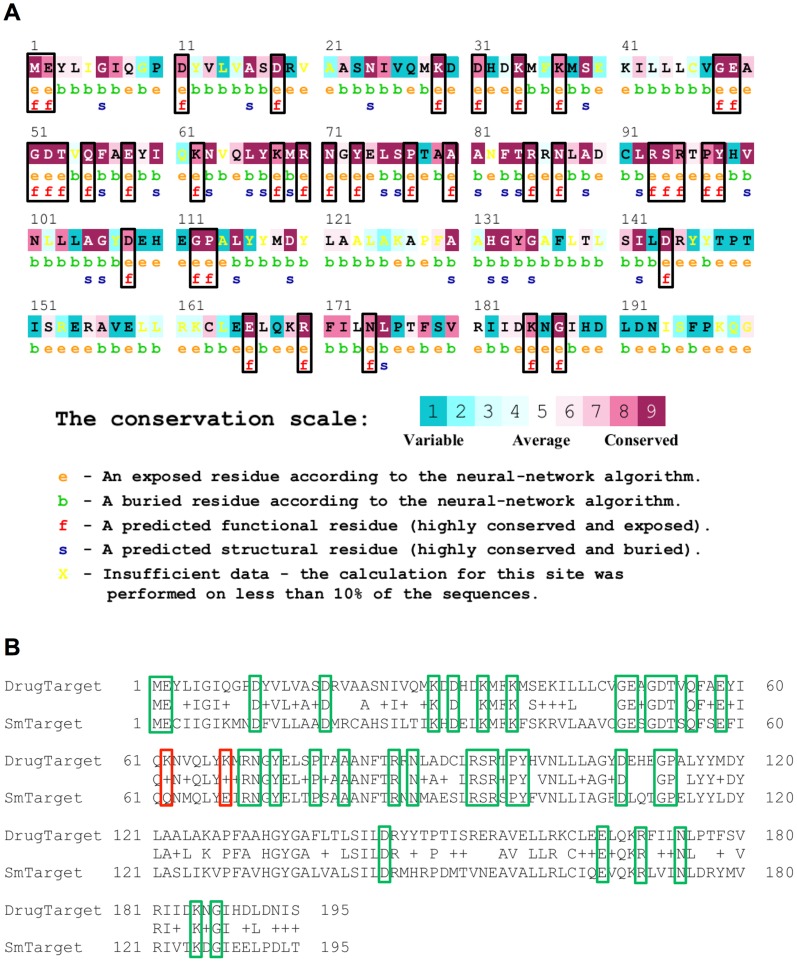
ConSurf analysis of the functional regions between an approved drug target and the corresponding *S. mansoni* target. (**A**) ConSurf predictions demonstrated on human proteasome β type 2 (Gene ID: PSMB2), using 150 homologues obtained from the UNIPROT database. The sequence of the query protein is displayed with the residue conservation scores at each site color-coded onto it. The color-coding bar shows the coloring scheme; conserved amino acids are colored bordeaux, residues of average conservation are white, and variable amino acids are turquoise. The residues of the query sequence are numbered starting from 1 to 199. The first row below the sequence lists the predicted burial status of the site (*i.e.* “b”– buried versus “e” – exposed). The second row indicates residues predicted to be structurally and functionally important: “s” and “f”, respectively. (**B**) Analysis of the functional regions conserved with the corresponding *S. mansoni* proteasome β type 2. The green rectangles represent the conserved functional residues and red rectangles represent non-conserved functional residues.

Pairwise sequence alignment was used to compare the *S. mansoni* targets previously identified with their approved homologous drug targets using BLAST alignment. Ten targets had less than an 80% overlap with their corresponding approved targets and were excluded from further analyses due to the improbability of both proteins sharing the same active site. Next, we performed sequence alignments and comparisons of functional regions for approved drug targets and predicted *S. mansoni* targets. This step allowed the identification of functionally relevant features and conserved residues necessary for catalysis and residues critical for binding. Fig. 3 shows an example of the ConSurf analysis of the functional regions between an approved drug target (human proteasome β type 2) and the corresponding *S. mansoni* target. Fig. 3A shows the predictions demonstrated on human proteasome β type 2 (Gene ID: PSMB2) using 150 homologues obtained from the UniProt database. This analysis revealed that 38 residues were predicted to be functionally important to the catalytic activity of the human enzyme. The functional regions of the human proteasome β type 2 were aligned to the respective *S. mansoni* orthologue. This comparison demonstrated that the active site predicted for *S. mansoni* proteasome β type 2 is conserved when compared to functional regions of its respective human target (Fig. 3B).

### Compilation of the “predicted targets list”

After running each of the *S. mansoni* protein sequences through the three databases, all proteins with negative results (negative hits) were excluded from further analyses, whilst predicted targets from each database were compiled into a single Excel file, hereafter called the “predicted targets list” (S3 Table). Each positive hit was examined further using BLAST pairwise alignment and the ConSurf server. This strategy resulted in a list of 39 potential druggable *S. mansoni* targets (∼1.8% of the interrogated targets) that could interact with 215 approved drugs. The DrugBank, STICH 3.1 and TTD databases exclusively predicted 120 (56.0%), 6 (2.8%), and 18 (8.3%) of the approved drugs, respectively, whilst the remaining 71(32.9%) drugs were predicted by two or three of these databases. Detailed information about the predicted targets and their associated drugs are provided in S3 Table. The distribution of the 39 identified *S. mansoni* targets according to their expression group is shown in Fig. 1B. We found that 19 (48.7%) of the predicted *S. mansoni* targets are in group III, 7 (17.9%) are in group I, 6 (15.4%) are in group II, and 7 (17.9%) are in group IV (Fig. 1B and S3 Table).

### List of drugs yet to be tested against *Schistosoma* species

To investigate which of the predicted drugs have already been tested against *Schistosoma* species, we undertook a literature search of PubMed, PubChem Bioassay, and SciFinder. A total of 22 druggable targets associated with 47 drugs whose activity has been previously evaluated against *Schistosoma* were identified. Examples of some of these drugs and their corresponding targets are given in Table 1. Accordingly, we were confident that our chemogenomic strategy for identifying new schistosomicidal drugs is valid. Consequently, we predicted 168 drugs to be active against 33 druggable targets that have not yet been experimentally tested against schistosomes or that have not yet required further studies. The results are summarized in Fig. 2. The complete list of predicted drugs, their targets, alignment parameters, and conservation of the functional regions is given in S3 Table.

**Table 1:** Examples of approved drugs and their potential *S. mansoni* targets that were previously reported on the literature, correctly identified by our target-based chemogenomics strategy.

Drug	Drug class	*S. mansoni* target (Target ID)	Biological process	Functional regions (%)	Activity data
methotrexate	antineoplasic	dihydrofolate reductase (Smp_175230.1)	synthesis of nucleic acid precursors	moderate conservation (78%)	IC_50_ = 7 nM enzymatic assay [109]
flubendazole	antihelminthic	tubulin α chain, putative (Smp_016780.1)	cytoskeleton formation	high conservation (100%)	79.5% of reduction of adult worms 25 days after infection (100 mg/kg) [110]
clonazepam	hypnotic and sedative	peripheral type benzodiazepine receptor (Smp_102510.1)	receptor activity	high conservation (83%)	IC_50_ = 10 µM in adult worms [111]
auranofin	antirheumatic	thioredoxin glutathione reductase (Smp_048430.1)	cell redox homeostasis	high conservation (92%)	IC_50_ = 0.7 nM enzymatic assay [112]
artesunate	antimalarial	Ca^2+^ transporting ATPase (Smp_136710.1)	Ca^2+^ homeostasis	high conservation (99%)	97.1% reduction of adult worms 30 days after infection (30 mg/kg) [113]
nifedipine	antihypertensive	voltage-dependent calcium channel (Smp_159990.1)	Ca^2+^ homeostasis	high conservation (88%)	Loss of motility and muscle contraction in adult worms (1 mg/kg) [82]

### Chemical space analysis

Finally, we used chemical space analysis to map the 168 predicted drugs in a multidimensional space using physicochemical descriptors for a dataset of active and inactive compounds against *Schistosoma* reported in the literature. The chemical space analysis is useful to refine the results and select the most promising drugs that share essential structural and physicochemical properties with schistosomicidal compounds. Because the original dataset was unbalanced, containing 355 active compounds *vs.* 331,228 inactive compounds (extracted from 101 bibliographic references, including 87 articles from PubMed and 14 from PubChem bioassays), it was not appropriate to build multivariate models [33]. For this reason, a balanced dataset containing 696 chemical structures (355 active compounds *vs.* 341 inactive compounds, S2 Table) was generated using the *k*NN method.

**Figure 4:**
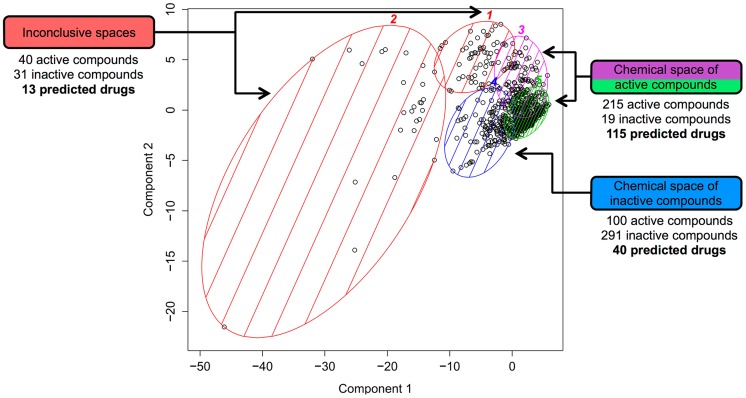
Chemical space analysis of the schistosomicidal compounds. Purple and green regions (clusters 3 and 5, respectively) represent the chemical space of active compounds. 115 drugs predicted by our strategy are inside this area and are more likely to be active. The blue region (cluster 4) represents the chemical space of inactive compounds and red region (clusters 1 and 2) are inconclusive spaces. 53 predicted drugs are inside this area and are more likely to be inactive. The first and second components explain 70.9% of the total variability.

The chemical space mapping was performed using *k*-means clustering via PCA using 39 physicochemical descriptors (Fig. 4). According to the PCA, the first and second principal components (PCs) explained 70.9% of the total variability of data and were categorized into five main clusters. Most of the compounds predicted to be active were located in regions marked in purple and green at the upper right corner of the score plot, totaling 215 active compounds (91.8%) and 19 inactive compounds (8.2%) (Fig. 4, clusters 3 and 5, respectively). Moreover, the inactive compounds were mostly delimited into the blue region containing 291 inactive compounds (74.4%) and 100 active compounds (25.6%) (Fig. 4, cluster 4). The red regions located in the center and at the upper right corner were flagged as inconclusive, as they contained similar proportions of both classes of compounds (Fig. 4, clusters 1 and 2, respectively). Remarkably, 115 drugs predicted by the proposed methodology are inside the overlapping area of the chemical space of the active compounds (Fig. 4, clusters 3 and 5) and are more likely to be active, whereas only 53 drugs were inside the overlapping area of the inactive compounds and the inconclusive clusters (Fig. 4, clusters 4, 1 and 2). Therefore, a “repurposing drug” located in the cluster regions 3 and 5 has a high probability (92%) of being active.

## Discussion

The main goal of this study was to identify drugs approved for clinical use in humans that may have potential schistosomicidal activity by performing a search of publicly available drug/target databases. However, since most target databases are only starting to be established, the predicted *S. mansoni* targets are not yet scored for druggability. The druggability concept adds a structural dimension and evaluates the likelihood that small drug-like molecules can bind a given target with sufficient potency to alter its activity [68]–[70]. Druggability is related to many factors, including the size of targets, the presence of pockets, and the overall charge and hydrophobicity of the interaction surface [68]. A number of factors were considered in this study in order to provide both confidence for the data generated and a solid basis from which to predict the druggability of individual *S. mansoni* targets. The predicted *S. mansoni* targets were considered druggable if they presented a sequence overlap ≥80%, a score ≥0.8, or an E-value ≤10^−20^ in relation to their predicted homologous targets and the high or moderate conservation of the functional regions. The overlapping sequences and analysis of functional regions among approved drug targets and *S. mansoni* targets revealed the importance of each position for the function of the predicted protein and the possible preservation of affinity for the predicted drug. Following this precondition, we were able to identify 168 drugs with the potential to inhibit their targets known to be transcribed in multiple life stages of *S. mansoni*. Moreover, in validation of the proposed chemogenomic strategy, several drugs previously demonstrated to be active against *Schistosoma* species in experimental assays were predicted by our methodology (Table 1 and S3 Table). Consequently, we were confident that our strategy for predicting schistosomicidal drugs is useful.

Additionally, we also evaluated the structural and physicochemical properties of known active and inactive schistosomicidal compounds to map the chemical space that is accessed by the 168 predicted drugs using the chemogenomics strategy. Chemical space is a term that is commonly used in place of “multi-dimensional descriptor space”, which is a region defined by a particular choice of descriptors encompassing all the possible compounds that could be mapped to the coordinates of this multi-dimensional space [71], [72]. This concept is closely related to the notion of chemical diversity. The diversity of a chemical library is a quantitative description of how different these compounds are from each other, with similar compounds and similar biological activities falling in the same chemical space region [73]. For this, we used *k*-means clustering via PCA, a method to compute the position of every compound in a two-dimensional coordinate system based on a set of computed properties. The PCA reduces high-dimensional data into a lower-dimensional space, thus making it more manageable and comprehensible by extracting essential information [74], [75]. Indeed, PCA transforms the original measured variables, such as physicochemical descriptors, into new uncorrelated variables called PCs, which are a linear combination of the original measured variables. As a result, we found that 115 drugs predicted by this chemogenomics strategy are inside the chemical space of active schistosomicidal compounds, yielding a higher degree of confidence in the predictions.

A previous chemogenomics screen in *S. mansoni* described by Caffrey et al. [47] identified 35 potential druggable targets for further investigation in drug discovery programs, showing the value of *in silico* approaches for drug discovery for schistosomiasis. Interestingly, only one drug target identified in that study (methionine aminopeptidase: Target ID  =  Smp_011120.1) is present amongst our predicted targets, which is likely because the studies differ significantly in their methodology. In addition, Berriman et al. [53] also reported an *in silico* approach to predict schistosomicidal drugs using the StARlite database and conservative parameters (≤50% sequential identity and ≥80% overlap) for target exclusion. Only eight predicted drugs identified in our study (carbidopa, colchicine, dasatinib, deserpine, mycophenolate mofetil, mycophenolic acid, reserpine and ribavirin) are overlapping in both studies. This small number might relate to the different gene datasets, different databases, and different parameters for conservation filtering used [76].

One of our goals was to predict targets that control the muscle function and motility of the parasite. Schistosomes depend on their muscular systems for motility, penetration of host skin (cercariae), and migration (schistosomules). Additionally, schistosomes use their muscular system for pairing and mating and reproductive, digestive, and excretory processes. These responses are essential for the survival of the parasite. These behaviors require precise coordination not only of the musculature that enables the movement but also of the responses regulated by the neurotransmitters needed for successful motility [77]. Drugs that disrupt one or more of these motility functions would be expected to interfere with the normal life of the parasite and would consequently cause its death. It is noteworthy that PZQ, the drug of choice to treat schistosomiasis, disrupts the muscle function and causes paralysis of the worm [78]. Currently, the gold standard test for measuring drug activity for *S. mansoni* is the *in vitro* assessment of worm motility, measured visually through microscopy [79]. Furthermore, we suggest that 61 drugs have potential activity against the muscle function and motility of the parasite because they were predicted to interact with 4 neurotransmitter transporters (nicotinic acetylcholine receptor α subunit, Target ID  =  Smp_031680.1; Na^+^/Cl^-^ dependent transporter, Target ID  =  Smp_193800.1; vesicular amine transporter, Target ID  =  Smp_121920.1; and Na^+^/Cl^-^ dependent neurotransmitter transporter, and Target ID  =  Smp_160360.1); 2 ion channels (Ca^2+^ transporting ATPase, Target ID  =  Smp_136710.1 and voltage-dependent Ca^2+^ channel, Target ID  =  Smp_159990.1); and 2 indirectly related proteins (calmodulin, Target ID  =  Smp_026560.2; and acetylcholinesterase, Target ID  =  Smp_154600.1) (S3 Table). These drugs are attractive when related to the study reported by Smout et al. [79] that can simply and objectively assess anthelmintic activity by measuring parasite motility in real time in a fully automated high-throughput screening.

Another important aspect considered in this study has to do with intellectual property protection of the potential schistosomicidal drugs predicted by the proposed strategy, particularly for those drugs that are off-patents. Pre-existing patents could impede the commercialization of schistosomicidal repositioned drugs and make them uneconomical, given that schistosomiasis predominantly affects poor populations in low- and middle-income countries. Therefore, an extensive search was done to collect the patent information (expiration date) of the predicted drugs using the European Patent Office database, Google Patents, and SciFinder. We found that 80.9% of the predicted drugs are off-patents (S3 Table). Last, we refer specifically to six drugs that we suggest are candidates for pre-clinical (*in vitro* and *in vivo*) studies (Table 2). The remaining drugs were not discussed in detail, because we found that pharmacokinetic and toxicity properties may render them less suitable as schistosomicidal drugs than the above chemicals. For example, ixabepilone and pralatrexate are antineoplastic drugs and thus might cause severe toxicity in humans. However, we consider that all predicted drugs identified in this study are attractive for further analysis.

**Table 2:** Examples of potential schistosomicidal drugs and their potential targets revealed in this study.

Drug	Therapeutic class	*S. mansoni* target (Target ID)	Biological process	Functional regions (%)
cinnarizine	anti-allergic	voltage-dependent calcium channel (Smp_159990.1)	Ca^2+^ homeostasis	high conservation (88%)
griseofulvin	antifungal	tubulin β chain, putative (Smp_192110.1)	cytoskeleton formation	high conservation (99%)
tetrabenazine	antipsychotic	vesicular amine transporter (Smp_121920.1)	neurotransmitter transport	moderate conservation (73%)
clotrimazole	antifungal	Ca^2+^-activated K^+^ channel (Smp_161450.1)	K^+^ homeostasis	high conservation (80%)
gentamicin	antibacterial	heat shock protein 73 (Smp_106930.1)	protein folding	high conservation (98%)
aprindine	antiarrhythmic	calmodulin, putative (Smp_134500.1)	Ca^2+^ binding messenger	high conservation (100%)

Cinnarizine is an antagonist of the histamine H1 receptor used for the control of nausea due to motion sickness. This drug is also considered a potent dilator of peripheral vessels because of its ability to block Ca^2+^ channels [80], [81]. The present study suggests that cinnarizine may also be able to inhibit the *S. mansoni* voltage-dependent L-type calcium channel subunit alpha-1S (Target ID  =  Smp_159990.1; E-value  = 0; functional regions  = 88% conservation), which is homologous to the human enzyme. Curiously, PZQ is considered a Ca^2+^ channel activator, which would allow more Ca^2+^ channels to open and lead to the disruption of normal intracellular Ca^2+^ levels. After exposure to PZQ, diverse effects become apparent in adult worms, such as muscular contraction and tegumental disruption, which subsequently leads to the exposure of parasite antigens on the worm's surface [78]. Despite the activator effect of PZQ on Ca^2+^ channels, a recent study demonstrated that treatment with nifedipine, a Ca^2+^ channel blocker, resulted in antischistosomal activity against schistosomula and significantly reduced their viability. Adult worms were also affected by nifedipine and exhibited impaired motility, several lesions on the tegument, intense contractility, and the reduction of egg deposition [82].

Griseofulvin is a fungistatic drug that is orally administered in the treatment of cutaneous mycoses. It was originally biosynthesized from *Penicillium griseofulvum* in 1939 [83], but its *in vivo* efficacy was first demonstrated only in 1958. Results from the present study suggest that griseofulvin might also be able to inhibit the *S. mansoni* tubulin-β chain (Target ID  =  Smp_192110.1; E-value  = 0; score  = 0.8; functional regions  = 99% conservation), which is homologous to the *Candida albicans* protein and is expected to be involved in cytoskeleton formation. Griseofulvin is able to inhibit the growth of fungal, plant, and mammalian cells by blocking the cells at the G2/M phase of the cell cycle [84], [85]. In fungi, griseofulvin deteriorates spindle and cytoplasmic microtubules, resulting in nuclear and mitotic abnormalities followed by distortions in hyphal morphology. Microtubules form a highly organized cellular cytoskeleton in eukaryotic cells, and their aggregation–disaggregation plays a key role in cell morphology and growth [86]. The concentration of griseofulvin required to deteriorate the spindle and cytoplasmic microtubules of fungal cells is much lower than that required to inhibit normal healthy mammalian cells due to its higher affinity for fungal tubulin as compared to mammalian tubulin [87]–[90]. Furthermore, griseofulvin selectively induces apoptosis in several cancer cell lines, sparing the normal healthy cells [85], [91]. Therefore, we consider that griseofulvin has low toxicity against normal healthy cells, which makes it highly appropriate for clinical use.

Tetrabenazine is a reversible human vesicular monoamine transporter type 2 inhibitor. It acts within the basal ganglia and promotes the depletion of monoamine neurotransmitters, such as serotonin and dopamine, in nerve terminals [92]. In this study, we suggest that tetrabenazine might also be able to block the vesicular amine transporter of *S. mansoni* (Target ID  =  Smp_121920.1; E-value  = 3.06^−130^; functional regions  = 73% conservation). *S. mansoni* also has a sophisticated nervous system that includes both central and peripheral elements and employs a wide range of neurotransmitter transporters. Among them, there are vesicular amine transporters that are normally responsible for the uptake of cytosolic biogenic amines into synaptic vesicles. Serotonin and dopamine are largely responsible for neuromuscular signaling in the parasite, and therefore, the carriers are expected to be important components of the worm's motor control system [77], [93]. It is worth noting that amine transport inhibitors have been shown to have strong effects on the parasite, as demonstrated in two medium-throughput drug screens of *S. mansoni* [94], [95].

Clotrimazole is an antifungal drug commonly used to treat yeast infections of the vagina, mouth, and skin, such as athlete's foot, jock itch, and body ringworm. This drug is a potent inhibitor of 14-α demethylase, resulting in increased cellular permeability. It is also capable of inhibiting the movement of Ca^2+^ and K^+^ ions across the cell membrane by blocking the Ca^2+^-activated K^+^ channel [96]. The present study suggests that clotrimazole might also be able to block the *S. mansoni* Ca^2+^-activated K^+^ channel (Target ID  =  Smp_161450.1; E-value  = 1.09^−48^; functional regions  = 80% conservation). The Ca^2+^-activated K^+^ channel is essential for maintaining the membrane in a hyperpolarized state, thereby regulating neuronal excitability, smooth muscle contraction, and secretion [97], [98]. Thus, the blocking of Ca^2+^-activated K^+^ channels in the muscle membranes of *S. mansoni* could be intimately involved in the dysfunction of rhythmic muscle activity. Due to its own chemical nature, clotrimazole is not well absorbed from the gastrointestinal tract. However, since clotrimazole is commercially available in powder form, it may be tested directly after dilution in an appropriate vehicle in experimental models of schistosomiasis with administration via a route other than oral, such as intra-peritoneal.

Gentamicin is an aminoglycoside drug composed of a mixture of related gentamicin components and is used to treat many types of bacterial infections, particularly those caused by gram-negative organisms. This drug binds the 30S subunit to the 16S ribosomal RNA (rRNA) of bacteria, but its affinity to the heat shock protein (HSP) 73 has also been well established [99]. We found that gentamicin might be able to interfere with the heat shock protein 70 of *S. mansoni* (Target ID  =  Smp_106930.1; E-value  = 0; functional regions  =  98% conservation), a homologue of the human HSP73. HSPs are a family of proteins involved in basic life-protecting mechanisms against harmful extracellular effects, primarily heat shock response. Normally, the expression of these proteins is increased in response to cellular adaptation to high temperatures [100]. Among the HSP family, HSP70 is considered the most predominantly conserved with intracellular chaperone and extracellular immunoregulatory functions [101]. In *S. mansoni*, it is well established that HSP70 is involved in protein re-folding and the chaperone function as an adaptive response to the rapid temperature rise between fresh water (∼ 28°C), in which the cercariae are found, and the warmer mammalian host (∼ 37°C) [53].

Last, we refer to aprindine as a candidate. This is possibly one of the least obvious drugs to hold schistosomicidal activity because it is not an anti-infective agent but rather an anti-arrhythmic drug. An interesting fact is that aprindine has a binding affinity to calmodulin [102]. Thus, we suggest that aprindine may also be able to inhibit the *S. mansoni* calmodulin (Target ID  =  Smp_026560.2; E-value  =  4.45^−81^; functional regions  =  100% conservation). Calmodulin is the primary sensor of intracellular Ca^2+^ levels that binds to and regulates a number of diverse target proteins involved in different functions, such as muscle contraction, apoptosis, and the immune response [103]. In *S. mansoni*, selective calmodulin inhibitors are known to disrupt egg hatching or cause miracidia to become vesiculated and die without undergoing transformation to the sporocyst stage [104], [105]. Ca^2+^ mobilization also plays a role in the cercarial penetration processes, possibly by calcium regulation of protease activities during infection [106], [107]. It is important to mention that Ca^2+^ ions are second messengers that are crucial for many biological functions, including muscle contraction, metabolism, and cell motility [108]. Importantly, visual inspection of the chemical structures allowed us to discover that aprindine is chemically similar (two aromatic centers and one aliphatic amine) to tricyclic drugs, a chemical class of the psychoactive drugs overactive against schistosomula stages [94].

Besides the drugs highlighted above, 109 other drugs are predicted to be active against *S. mansoni*. In all cases, we considered the numerical parameters (overlap, conserved functional regions, E-value, and score) for target homology sufficiently significant to infer drug predictions. Moreover, we verified that these drugs are “inside the chemical space” of active schistosomicidal compounds, making the predictions more reliable. Therefore, all 115 predicted drugs are candidates for drug repositioning and might be used as starting points for further *in vitro* and *in vivo* studies and schistosomicidal drug design because they are privileged structures and have established pharmacokinetic and toxicity profiles considered suitable for lead optimization.

### Conclusions

We used an *in silico* drug repositioning strategy based on the concept that “similar targets have similar ligands” to compile a list of drugs with potential activity against schistosomes. In doing so, we predicted 115 such compounds that we suggest justify evaluation as schistosomicidal drugs. We recognize that the activity of such compounds might be affected by appropriate chemical affinity with their predicted target. However, in addition to previous strategies, we used the criterion of conservation of functional residues among *S. mansoni* and its homologous targets and investigated the chemical space of known schistosomicidal compounds to further increase confidence in our predictions. Primary *in vitro* screens with these drugs might provide insights into their schistosomicidal activity. If promising activities are discovered, they could constitute important starting points for lead identification and optimization.

## Supporting Information

S1 Fig **Workflow of the chemical space analysis using the KNIME graphical user interface.** All steps of dataset balancing, processing, and chemical space analysis were implemented in R and KNIME, a graphical user interface that allows assembly of nodes for modeling, data analysis, and visualization.
(TIF)

S1 Table **List of** ***S. mansoni*** **genes with deferential expression in multiple life stages.**
(XLSX)

S2 Table **Dataset of known schistosomicidal compounds, with SMILES line notation for describing chemical structures, references, molecular descriptors and cluster analysis results.**
(XLSX)

S3 Table **List of predicted schistosomicidal drugs and target information.**
(XLSX)
