# Correction: *In Silico* Repositioning-Chemogenomics Strategy Identifies New Drugs with Potential Activity against Multiple Life Stages of *Schistosoma mansoni*

**DOI:** 10.1371/journal.pntd.0003554

**Published:** 2015-02-18

**Authors:** The PLOS Neglected Tropical Diseases Staff

The Funding section is incorrect. The correct funding is as follows: BJN was supported by a fellowship from the Coordination for the Improvement of Higher Education Personnel (CAPES). This work has been funded by the National Counsel of Technological and Scientific Development (CNPq) and the State of Goias Research Foundation (FAPEG). The funders had no role in study design, data collection and analysis, decision to publish, or preparation of the manuscript.
