# Supplementary figures and images for: In Silico Repositioning-Chemogenomics Strategy Identifies New Drugs with Potential Activity against Multiple Life Stages of Schistosoma mansoni
Source: PLoS Negl Trop Dis. 2015 Jan 8;9(1):e3435. doi: 10.1371/journal.pntd.0003435 (PMC4287566; doi:10.1371/journal.pntd.0003435)

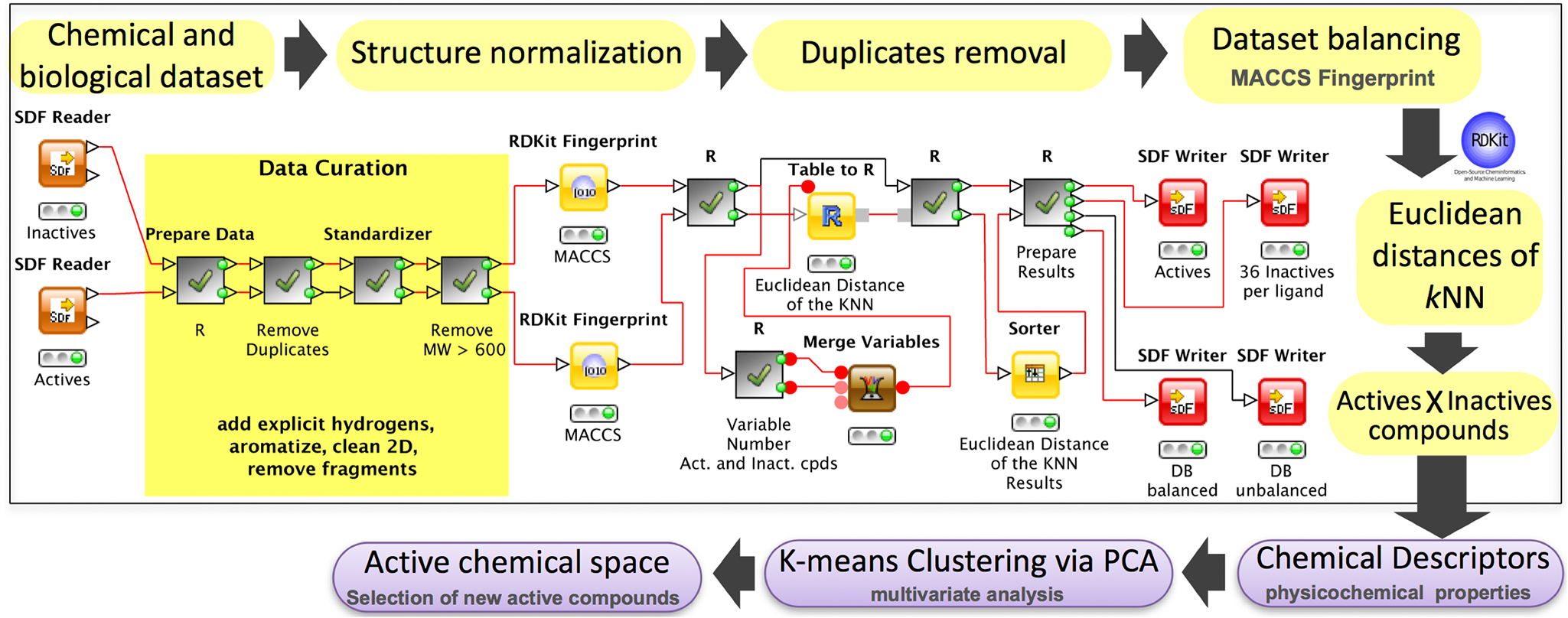

Supplement: S1 Fig — Workflow of the chemical space analysis using the KNIME graphical user interface. All steps of dataset balancing, processing, and chemical space analysis were implemented in R and KNIME, a graphical user interface that allows assembly of nodes for modeling, data analysis, and visualization. (TIF) [file pntd.0003435.s001.tif]
